# Pure secretory carcinoma in situ: a case report and literature review

**DOI:** 10.1186/s13000-019-0872-7

**Published:** 2019-08-23

**Authors:** Ying Yang, Zhiyuan Wang, Guoqing Pan, Shumo Li, Yingying Wu, Liu Liu

**Affiliations:** 1 Department of Pathology, The First Affiliated Hospital of Kunming Medical University, 295, Xichang Road, Kunming, 650032 Yunnan China; 2 Department of Breast Surgery, The First Affiliated Hospital of Kunming Medical University, 295, Xichang Road, Kunming, 650032 Yunnan China; 3 Department of Plastic Surgery, The First Affiliated Hospital of Kunming Medical University, 295, Xichang Road, Kunming, 650032 Yunnan China

**Keywords:** Breast, Secretory carcinoma, Carcinoma in situ, Diagnosis and differential diagnosis, *ETV6-NTRK3*

## Abstract

**Background:**

Secretory breast carcinoma is an exceptionally rare type of breast carcinoma. Only 5 cases of pure secretory carcinoma in situ have been reported in English literature. Herein, we reported a rare case of pure secretory breast carcinoma in situ.

**Case presentation:**

The patient is a 38-year-old female with bloody discharge from the left nipple. Microscopically, the terminal-duct lobular units were enlarged and filled with tumor cells. The tumor cells were arranged in cystic, microcystic, solid and papillary pattern and formed a honeycomb-like appearance. The presence of intracellular and extracellular eosinphilic PAS-positive material was the most remarkable feature. Immunohistochemically, myoepithelial markers highlighted the complete presence of myoepithelial cells around the tumour nests. Tumour cells were strongly positive for S-100 and CK5/6, negative for ER, PR and HER2. Fluorescence in situ hybridization analysis showed *ETV6-NTRK3* fusion.

**Conclusion:**

Secretory carcinoma in situ shares the same morphological, immunohistochemical and molecular features with invasive secretory carcinoma except that the papillary growth pattern is more common in the introductal components. Cautions should be taken to distinguish secretory carcinoma in situ from other introductal lesions. Our report is an important supplement to the morphology spectrum of secretory breast carcinoma.

## Background

Secretory breast carcinoma (SBC) is an exceptionally rare type of breast carcinoma, accounting for less than 0.15% of all breast cancers [1]. Secretory breast carcinoma elicits pathological interests because of its unique morphology, characteristic molecular alteration, basal-like immunophenotype, and a favorable prognosis. Although in situ secretory carcinomas are usually reported to be present with invasive components, no research has focused on this precursor lesions of SBC. Only 5 cases of pure secretory carcinoma in situ have been reported in the English literature to our knowledge. Herein, we reported a rare case of pure secretory breast carcinoma in situ of a 38-year old female.

## Case presentation

A 38-year-old Chinese female was admitted to our hospital with bloody discharge from the left nipple for 20 days. There was no special medical history or family history of any type of tumor. No mass was observed on physical examination. Breast sonogram showed dilatation in several ducts of the left breast, with the widest diameter of 0.4 cm. There was solid component located in the dilated duct indicating intraductal papillary lesion. Color Doppler sonography revealed no blood flow signals within it. Sonographic assessment was classified as Breast Imaging Reporting and Data System category (BI-RADS) 4a. A duct-lobular segmentectomy was performed. After the diagnosis of secretory carcinoma in situ was confirmed, the patient received mastectomy and sentinel lymph node biopsy. No evidence of metastasis was found in the 5 sentinel lymph nodes. The patient received no chemotherapy or radiotherapy, and remains free of local-regional recurrence or distant metastases after 13 months’ follow-up.

**Fig. 1:**
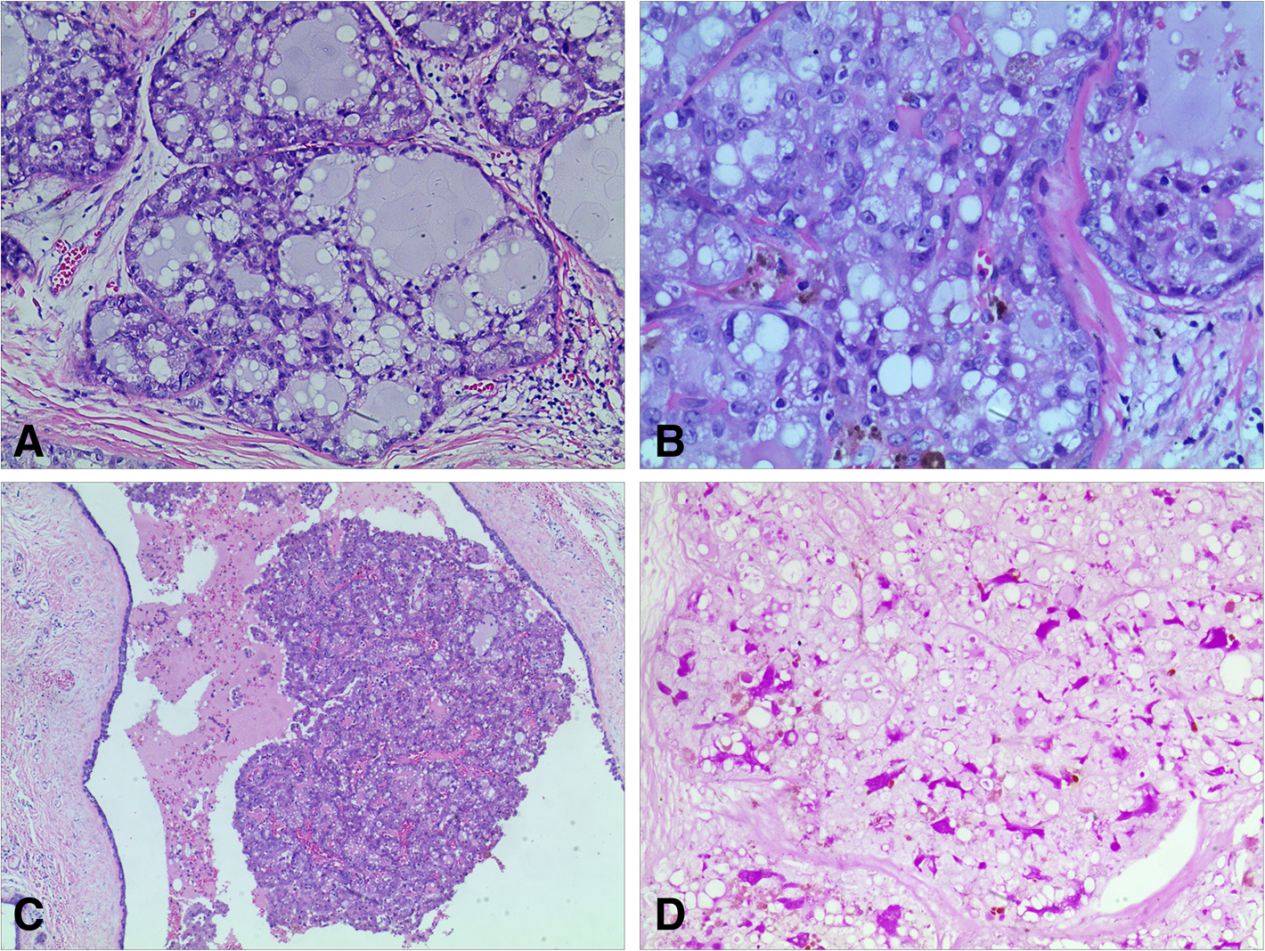
The microscopic character of secretory carcinoma in situ. **a**, The tumor cells were arranged in cystic and microcystic pattern, and formed a honeycomb-like appearance (HE, × 100). **b**, Tumor cells were mild-to-moderate atypia, with granular or vacuolated cytoplasm and vesicular nuclei containing small nucleoli (HE, × 200). **c**, In some areas, tumor cells were arranged in a papillary pattern within an dilated duct (HE, × 40). **d**, PAS-positive material in ductal lumina and in intracytoplasmic vacuoles (PAS, × 100).

Grossly, the tissue was irregular and non-encapsulated. The cut section was greyish white to yellow. No demarcated nodule was seen. Microscopically, the terminal-duct lobular units were enlarged and filled with tumor cells. The tumor cells were arranged in cystic, microcystic and solid pattern and formed a honeycomb-like appearance (Fig. 1A). The presence of intracellular and extracellular eosinphilic material was the most remarkable feature (Fig. 1B). In other areas, tumor cells were arranged in a papillary pattern with multiple layers of tumor cells and delicate fibrovascular core within an dilated duct (Fig. 1C). The intracellular and extracellular eosinphilic material was also predominant. Both the intracellular and extrocellular secretory material was positively stained by periodic acid-Schiff stain (PAS) (Fig. 1D). Tumour cells were mild to moderate atypia with pale to eosinphilic, foamy or vacuolated cytoplasm. Nuclei were round-to-oval with or without a small nucleolus. Mitotic activity was rare. No necrosis or invasive component was present. In the specimen of mastectomy, no invasive or in situ carcinoma was found.

**Fig. 2:**
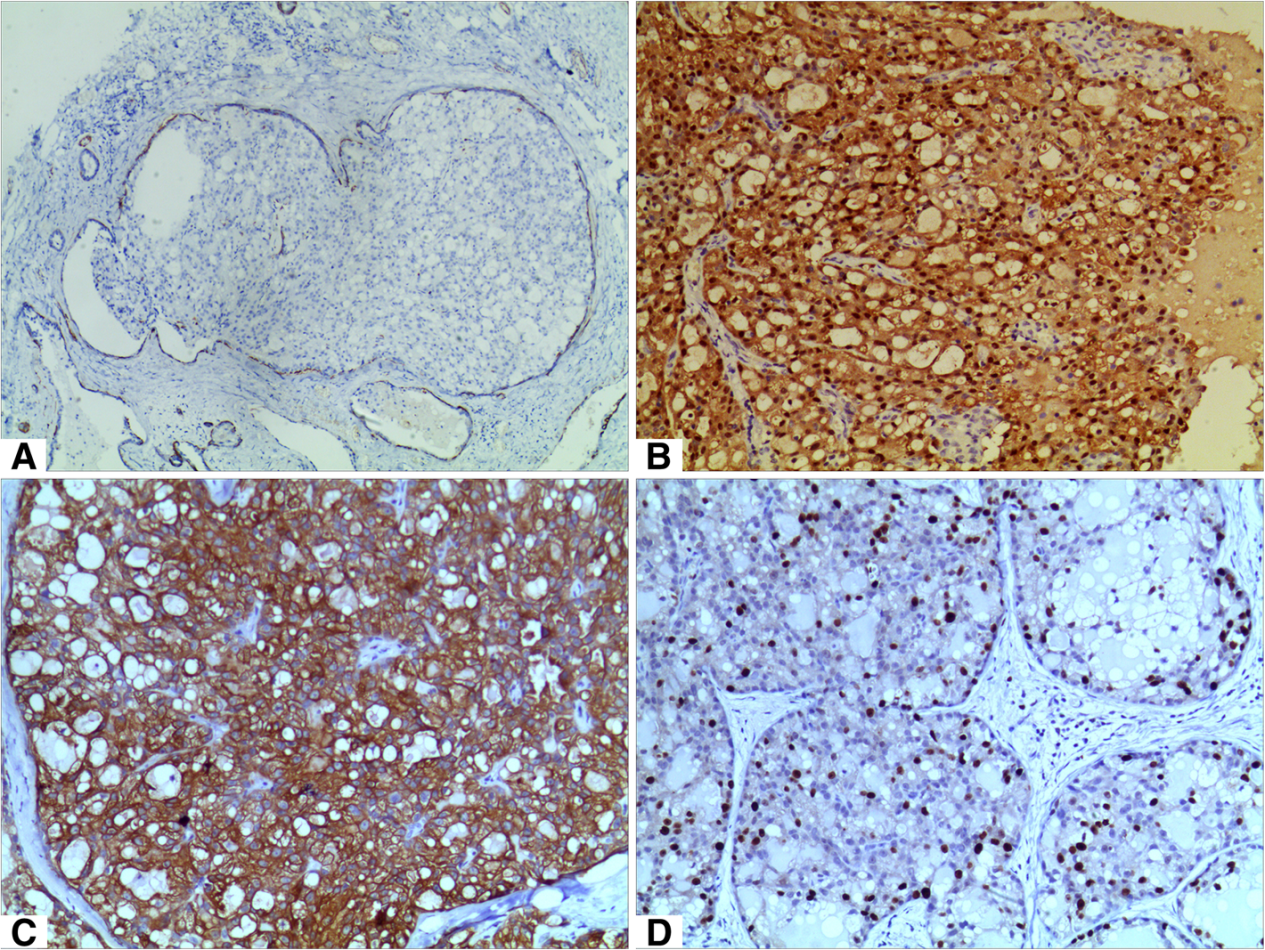
The immunohistochemial character of secretory carcinoma in situ. **a**, SMMHC highlighted the complete presence of myoepithelial cells around the tumour nests (Envision, × 40). **b**, Tumor cells were diffusely positive for S100 (Envison, × 100). **c**, Tumor cells were diffusely positive for CK5/6 (Envison, × 100). **d**, The Ki-67 index was about 10% (Envison, × 100)

Immunohistochemically, myoepithelial markers including P63, smooth muscle myosin heavy chain (SMMHC) and calponin highlighted the complete presence of myoepithelial cells around the tumour nests and the dilated ducts (Fig. 2A). Tumour cells were diffusely positive for S-100 (Fig. 2B), CK5/6 (Fig. 2C), pan-CK, CK7 and GATA3, and negative for oestrogen (ER), progesterone receptor (PR) and human epidermal growth factor receptor 2 (HER2). Ki67 index was 10% (Fig. 2D).

**Fig. 3:**
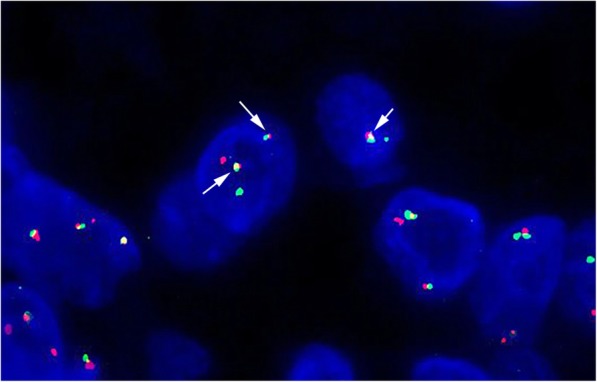
Fluorescence in situ hybridization analysis of the secretory carcinoma in situ. The fused red and green signals (arrows) indicate the presence of *ETV6-NTRK3* fusion genes

Fluorescence in situ hybridization (FISH) analysis was performed using *ETV6/NTRK3* fusion translocation t (12;15) probe (LBP Medicine Science and Technology, Guangzhou, China). The red signal represents *ETV6*, and the green signal represents *NTRK3*. Tumour cells were shown to have rearrangement of *ETV6* gene (Fig. 3), with an increase number of *ETV6-NTRK3* fusion signals (32.5%) above the cut-off value (10%).

## Discussion

Secretory breast carcinoma was first reported as “juvenile breast carcinoma” by McDivitt and Stewart in 1966 [2], and was renamed *secretory carcinoma* in the 1980s by Tavassoli [3] because additional cases were reported in adults. It was considered one of the exceptionally rare types of breast carcinomas. SBC has been reported in both sexes and the reported male-female ratio was 1:6. The median age of presentation is 25 years (range, 3–87 years) [1, 4]. The characteristic pathological morphology is a solid and microcystic architecture composed of low-grade tumour cells that produce intracellular and extracellular secretory material that is positive on PAS stain. Immunohistochemically, the tumour cells show consistently positive for S-100 and α-lactalbumin.

Although intraductal components usually accompany with invasive SBC [5], no research has focused on the in situ lesions of SBC. We reviewed the previous studies of SBC from 1990 to 2018; intraductal secretory carcinoma with invasive components was described in several studies [6–8], with or without specific description of in situ components. Only five cases of pure secretory carcinoma in situ have been reported to our knowledge [9–11], including three cases in a research investigating the expression of STAT 5a which had no clinicpathological details except ages and genders [11], and the significance of this uncommon lesion was not discussed further. Our report is an important supplement to the morphology spectrum of SBC.

The clinical and pathological features of the six cases of secretory carcinoma in situ (including the current case) were reviewed in Table 1. Although most SBC patients were juvenile or young adults (and this is why it was named *juvenile breast carcinoma* when first reported), the median age of the cases of pure secretory carcinoma in situ was 48.5 years (rang, 30–73 years). The male-female ratio was 1:5. The clinical presentation included lump and bloody nipple discharge. Histologically, microcystic and cribriform pattern containing PAS-positive secretory material remained as the predominant feature in the dilated ducts. Papillary growth pattern presented in all the three cases that have described the morphological features although it was an unusual morphology in its invasive counterpart [12]. Immunohistochemically, in the three cases which underwent immunohistochemistry, two cases expressed ER, and one case (our case) was triple-negative. Consistent with the invasive SBC in the literature, tumor cells of our case showed defuse positive staining for S-100 and the basal-like marker CK5/6 [13]. In 2002, Tognon et al. reported that secretory breast carcinoma are associated with a characteristic balanced translocation, t (12;15), that creates an *ETV6/NTRK3* gene fusion [14]. In all the six cases of secretory carcinoma in situ, *ETV6/NTRK3* fusion was only identified in our case. These results are concordant with those of Lae et at [15]: in situ and invasive components had the same immunoprofile and molecular features, highlighting their genetic similarities.

**Table 1:** Reported cases of pure secretory carcinoma in situ

Author	Sex	Age (yo)	Symptoms/ Duration (month)	Site/Location	Histological growth pattern	Treatment	HR	HER2	*ETV6-NTRK3*	Axillary status	Follow-up(m)
Kameyama et al [9]	M	51	Lump/ND	L/Subareolar	Papillary, cribriform	RM	ER+	ND	ND	-(0/?)	NED
Sato et al [10]	F	30	Lump/6	L/Lower inner	Papillary, cribriform	SM + SLNB	ER+,PR-	–	NE	-(0/2)(SLNB)	NED 60
Strauss et al [11]	F	73	ND	ND	ND	ND	ND	ND	ND	ND	ND
	F	51	ND	ND	ND	ND	ND	ND	ND	ND	ND
	F	46	ND	ND	ND	ND	ND	ND	ND	ND	ND
Our case	F	38	Bloody nipple discharge/1	L	Microcystic, solid,papillary	RM + SLNB	ER-,PR-	–	+	-(0/5)(SLNB)	NED 13

Abbreviations: ER, estrogen receptor; F, female; HER2, human epidermal growth factor receptor 2; HR, hormone receptor; L, left breast; M, male; ND, not defined; NE, not examined; NED, not evidence of disease; PR, progesterone receptor; RM, radical mastectomy; SLNB, sentinel lymph node biopsy; SM, simple mastectomy;

The differential diagnosis of secretory carcinoma in situ include lactational change, cystic hypersecretory hyperplasia, cystic hypersecretory carcinoma, juvenile papillomatosis with apocrine metaplasia, apocrine carcinoma in situ, and lobular carcinoma in situ. Lactational change differ from secretory carcinoma in situ in its diffuse change involving the whole breast, totally bland cell morphology, and lack of complicated intraductal structure. The epithelium lined in the cysts of cystic hypersecretory hyperplasia are cubboidal or columnar that resembling thyroid follicle. Most cases of cystic hypersecretory carcinoma are intraductal carcinomas that may be confused with SBC in situ. However, unlike cystic hypersecretory carcinoma, secretory carcinoma in situ contains only focal areas of cystic formation and produces more bubbly secretions. Apocrine cytologic features include prominent eosinophilic, flocculent or granular cytoplasm, sharply defined cell borders and large nuclei containing prominent nucleoli. The nuclear grade is usually high in contrast to the low grade nuclei of secretory carcinoma, and no intra and extracellular secretory material was found. On the other hand, about one half of apocrine carcinoma exhibit HER2 overexpression, whereas most secretory carcinomas are HER2-negative. The intracytoplasmic mucin of lobular carcinoma is not as abundant as in secretory carcinoma, and there are no extracellular secretory material characteristic of secretory carcinoma. The unique *ETV6/NTRK3* fusion provides a further way to distinguish SBC from other breast tumors. Besides, cautions should be taken to avoid underdiagnosing secretory carcinoma in situ as usual ductal hyperplasia (UDH) because of its diffuse expression of CK5/6. SBC in situ can be easily differentiated from UDH by morphological observation.

Although secretory breast carcinoma belongs to the basal-like carcinoma spectrum, it has immunohistochemical and genetic features that distinguish them from other basal-like tumors of the breast [15]. Recently, Jin et al. revealed that SBC shares genomic mutations and biological pathways more closely related to hormone receptor-positive breast cancer than basal-like triple-negative breast cancer by exome sequencing and proteomic analysis of SBC [16]. Overall, the patients with secretory carcinoma have a favorable prognosis with a 5-year overall survival of 87.2% [17]. Features that ensure an excellent prognosis include young age, small tumour size (under 2 cm), and the absence of stromal invasion at the periphery of the lesion [18]. The study of Castillo et al. pointed out that even in proven SBC, histologic grade and TNM stage overruled for prognosis and therapeutic management [19]. For the three cases of pure secretory carcinoma in situ that had recorded the clinical details, no axillary or sentinel lymph node was involved, and there was no evidence of recurrence or metastasis in the 8–60 months follow-up.

Surgery is still considered the most appropriate treatment for SBC, but there is no consensus about the extent of surgery. Conservative surgery or simple mastectomy with sentinel lymph node biopsy has been chosen by most surgeons for patients of invasive SBC [20]. The real value of postoperative radiotherapy and chemotherapy has not been established. More recently, successful targeted therapy of refractory *ETV6-NTRK3* fusion-positive SBC in a 14-year-old girl has drawn wide attention [21]. The almost immediate and extraordinary response to the TrK inhibitor larotrectinib gave a textbook example of precision medicine. To date, there are limited published data on the biological behavior and long-term clinical outcome of pure secretory carcinoma in situ, so it is necessary for the patients to be closely followed-up.

## Conclusion

In this paper, we report a case of pure secretory breast carcinoma in situ of a 38-year-old female on the basis of imaging, histopathological pattern, immunophenotype and molecular alteration. Secretory carcinoma in situ shares the same morphological, immunohistochemical and molecular features with invasive SBC except that the papillary growth pattern is more common in the introductal components. Cautions should be taken to distinguish secretory carcinoma in situ from other introductal lesions.

## Data Availability

Not applicable.

## Abbreviations

BI-RADS: Breast Imaging Reporting and Data System
ER: oestrogen
FISH: Fluorescence in situ hybridization
HER2: Human epidermal growth factor receptor 2
PAS: Periodic acid-Schiff stain
PR: Progesterone receptor
SBC: Secretory breast carcinoma
SMMHC: Smooth muscle myosin heavy chain
UDH: Usual ductal hyperplasia
